# Chemical Textures on Rare Earth Carbonates: An Experimental Approach to Mimic the Formation of Bastnäsite

**DOI:** 10.1002/gch2.202400074

**Published:** 2024-05-17

**Authors:** Melanie Maddin, Remi Rateau, Adrienn Maria Szucs, Luca Terribili, Brendan Hoare, Paul C. Guyett, Juan Diego Rodriguez‐Blanco

**Affiliations:** ^1^ Department of Geology School of Natural Sciences Trinity College Dublin Dublin 2 D02 PN40 Ireland

**Keywords:** cerianite, crystallization, hydroxylbastnasite, kozoite, rare‐earth‐carbonates

## Abstract

The interaction between multi‐component rare earth element (REE) aqueous solutions and carbonate grains (dolomite, aragonite, and calcite) are studied at hydrothermal conditions (21–210 °C). The effect of ionic radii of five REEs (La, Ce, Pr, Nd, Dy) on solid formation are analyzed using two solution types: equal REE concentrations and concentrations normalized to Post Archean Australian Shale Standard (PAAS). The interaction replaces the host Ca–Mg carbonate grains with a series of REE minerals (lanthanite → kozoite → bastnäsite → cerianite). At 165 °C, equal concentration solutions promote kozoite crystallization, maintaining similar REE ratios in solids and solution. PAAS solutions result in zoned REE‐bearing crystals with heterogeneous elemental distributions and discreet REE phases (e.g., cerianite). Chemical signatures indicate metastable REE‐bearing phases transforming into more stable polymorphs, along with symplectite textures formed by adjacent phase reactions. Overall, experiments highlight the dependence of polymorph selection, crystallization pathway, mineral formation kinetics, and chemical texture on REE concentrations, ionic radii, temperature, time, and host grain solubility.

## Graphical abstract

The researchers investigate the interaction between rare earth elements (REEs) and Ca–Mg carbonate minerals in controlled environments. Through varying REE concentrations and temperatures, they observed intricate mineral formation processes. Their findings highlight the impact of REE concentrations, temperature, and mineral solubility on the selection of mineral polymorphs and the distribution of elements within the rock.

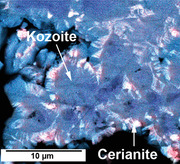

## 1 Introduction

As rare earth elements (REE) are essential in many green technologies, they play a critical role in a more sustainable future. However, there is a substantial risk to their supply as the availability of REE deposits with minable concentrations are limited. A better understanding of the mechanisms controlling REE concentration in minerals would have applications in more efficient mining practices as well as REE separation techniques and recycling.

REES are critical materials with their applications spanning a wide range including hybrid vehicles, rechargeable batteries, and wind turbines, as well as medical and military technologies.^[^ ^1^, ^2^ ^]^ With increased demand from the green energy market as well as REE applications migrating into common domestic items such as computer parts and television screens, the pressure is building on the current global REE supply chain. Presently, REE processing is dominated by China (≈85%).^[^ ^3^ ^]^ However, in recent years, a significant reduction in export quotas from China is leading to supply chain instability.^[^ ^4^ ^]^

REE deposits can be categorized as either primary or secondary deposits. Magmatic, hydrothermal, and/or metamorphic processes are responsible for the formation of primary deposits which are generally associated with alkaline igneous rocks and carbonatites. Some examples of these types of deposits include Mountain Pass, California and China's Bayan Obo, which is the world's largest REE deposit. In secondary deposits, formation occurs as a consequence of weathering and erosion, such as placers, laterites, and bauxites.^[^ ^5^ ^]^ For example, the weathering of tin granite in south China has resulted in REE‐laterite. Also, the humid, subtropical climatic conditions in northeast India have also promoted the formation of a thick laterite profile offering a promising alternative for this type of REE deposit.^[^ ^6^ ^]^ Presently, the world reserves of REE amount to ≈130 million tonnes and are under the control of China, Brazil, Vietnam, Russia, and India.^[^ ^7^ ^]^ However, China holds one‐third of the world's REE reserves and dominates in REE exploration and production. Bastnäsite, monazite, and xenotime are currently the principal economic resources of REE minerals and can be sourced from these geological deposits.^[^ ^8^ ^]^

In our study, we focussed on bastnäsite as it is one of the major sources of REE found in commercial deposits such Bayan Obo, China.^[^ ^9^ ^]^ Three natural carbonate‐fluoride minerals comprise the bastnäsite family each with varying amounts of REE. These include (Ce)‐bastnäsite; (Ce,La)[CO_3_]F, which is the most abundant, (La)‐bastnäsite; (La,Ce)[CeO_3_]F, and (Y)‐bastnäsite; (Y,Ce)[CO_3_]F. Synthetic bastnäsites are gaining increased attention as they can be used as precursors for rare earth oxycarbonate (RE_2_O_2_CO_3_) and oxyfluorides (REOF) synthesis, both of which can be utilized as host lattices for phosphors. (Ce)‐bastnäsite together with the phosphate mineral (Ce)‐monazite (Ce,La,Nd,Th)[PO_4_], make up our major source of Ce as well as additional REE such as La, Nd, and Y.^[^ ^10^ ^]^

When studying natural ore deposits, researchers will generally only characterize the final mineralogical assemblage. However, the aim of this study is to better understand the reaction pathways and crystallization sequence that leads to some rare earth carbonates. A better understanding of the mechanisms controlling the REE concentration in minerals would have applications in more efficient practices as well as separation techniques and recycling. Therefore, we have mimicked natural systems by carrying our replacement experiments using multi‐component REE aqueous solutions and carbonate host grains of dolomite, aragonite and calcite, which are commonly found in carbonatite ore deposits. All of the products obtained in our experiments were characterized with high resolution electron microscopy and powder X‐ray diffraction, allowing us to show that temperature as well as aqueous chemistry are key in controlling the mineralogy and distribution of REE in carbonate rocks.

## 2 Results

The analysis of all solid samples obtained from the replacement experiments resulted in the formation of a series of surface precipitates that partially or totally replaced the host carbonate grain. The combination of powder X‐ray diffraction (XRD), scanning electron microscopy (SEM) and energy dispersive X‐ray spectroscopy (EDS) allowed us to identify and quantify the newly formed phases and interpret the mechanisms responsible for some of the chemical textures exposed in the polished resin pucks.

### 2.1 Powder X‐ray Diffraction

**Figure 1:**
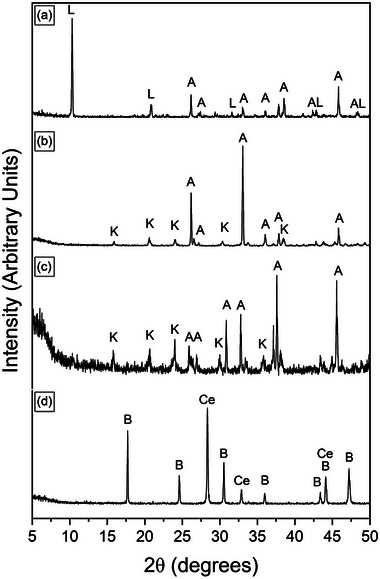
(Powder XRD patterns of lanthanite (L), kozoite (K), hydroxylbastnäsite (B) and cerianite (Ce) obtained from experiments reacting a) aragonite at 21 °C (non‐PAAS), b) 80 °C (non‐PAAS), c) 80 °C (PAAS) and (d) dolomite at 165 °C (PAAS.

**Figure 2:**
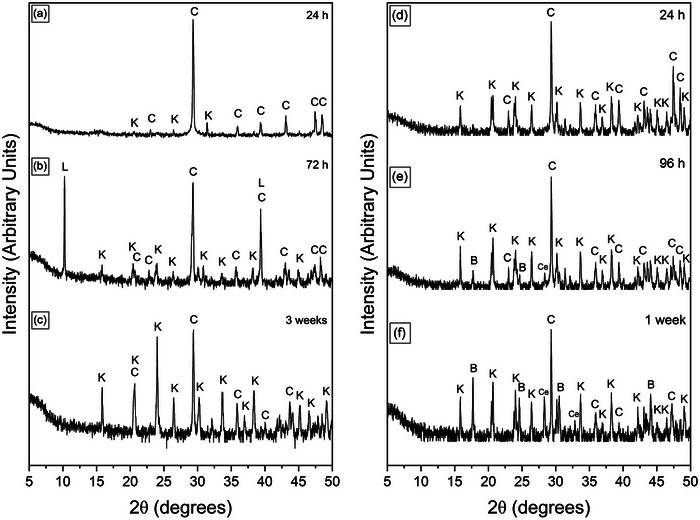
Powder XRD patterns of calcite in PAAS solution at 80 °C a) 24 h, b) 72 h, c) 3 weeks, and 165 °C, d) 24 h, e) 96 h, f) 1 week, *calcite (C), kozoite (K), bastnäsite (B)]. The extent of the replacement reaction was found to be time and temperature dependent, with 40% of calcite consumed after 3 weeks at 80 °C (c), and 37% of calcite consumed after just one week (f) at 165 °C.

The interaction of calcite, dolomite and aragonite with muti‐component rare earth element (La, Ce, Pr, Nd, and Dy) ‐bearing aqueous solutions resulted in the formation of secondary REE‐bearing minerals. The characterization with powder X‐ray diffraction (XRD) revealed the formation of a series of rare‐earth bearing carbonate minerals: lanthanite (REE_2_(CO_3_)_3_∙8H_2_O), kozoite (REE(CO_3_)(OH), hydroxylbastnäsite ((REE(CO_3_)(OH,F) (**Figure** **1**) and a REE oxide, cerianite (CeO_2_). These newly formed phases presented as surface precipitates that partially replaced the host mineral. In some experiments, the host carbonate minerals were fully replaced. The extent of the replacement reaction was found to be temperature and time dependent (**Figure** **2**). At certain temperatures, the formation of specific crystalline polymorphs was hindered during reaction. Lanthanite was found to be present and stable at low temperature only (21 °C), with minor amounts found in the aragonite experiments at 50 °C. Kozoite precipitated and remained stable at 50 and 80 °C. At 165 and 205 °C, kozoite also formed but it quickly transformed to hydroxylbastnäsite and cerianite. At 205 °C, hydroxylbastnäsite and cerianite precipitated after short reaction times (i.e., within 24 h). In some cases, the host mineral (e.g., calcite) was fully consumed by cerianite and/or hydroxyl‐bastnäsite. Cerianite was the end product of the higher temperature experiments, replacing the host and the REE carbonates. The formation of the REE carbonates in the reaction sequence was also found to relate to the nature of the host mineral (e.g., at 165 °C, hydroxylbastnäsite and cerianite precipitated faster in the aragonite experiments compared to the dolomite and calcite experiments). The REE concentration in the initial solution also played a key role, an example being the 21 °C experiments with lanthanite precipitating in the Post Archean Australian Shale (PAAS) solution experiments for all three host grains, however, in the non‐PAAS experiments, lanthanite only formed on the surface of aragonite, with no additional phases being detected by XRD on the surface of calcite or dolomite. There was a small amount of lanthanite precipitated in the aragonite experiments at 50 °C in the PAAS solution, compared to only kozoite in the dolomite and calcite experiments. At 80 °C, kozoite was the sole main stable phase in both the non‐PAAS and PAAS experiments. At 165 and 210 °C, kozoite quickly transformed to hydroxy‐bastnäsite and cerianite, with only slightly prolonged stability in the non‐PAAS solutions with dolomite and calcite grains. Also, at 165 and 210 °C, the amount of cerianite that formed in the non‐PAAS solution ranged from <1% to 5%, while in the PAAS solutions, Ce was the most highly concentrated element and resulted in cerianite replacing the host by 17–46%.

#### 2.1.1 Scanning Electron Microscopy–Energy Dispersive Spectroscopy (SEM–EDS)

**Figure 3:**
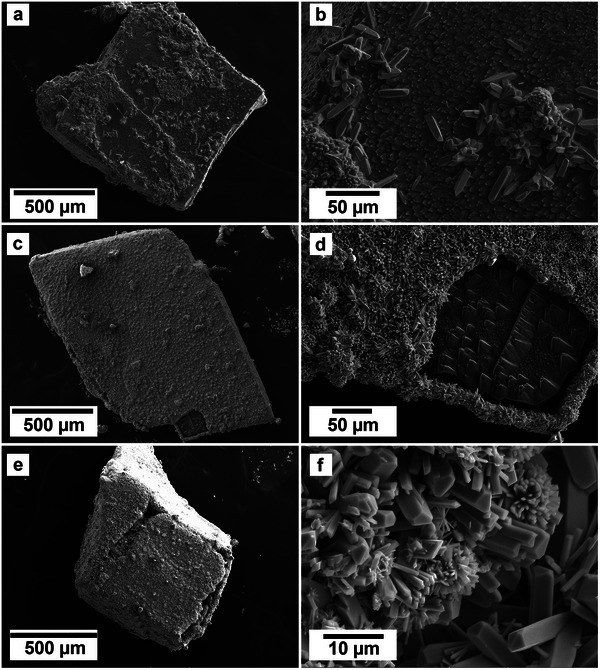
Secondary electron SEM images of the surface of calcite grains reacted with the PAAS solution at 80 °C showing the progressive replacement by REE carbonates (left: grain view, right: detailed view), after a,b) 24, c,d) 72 h, and e,f) 4 weeks.

**Figure 4:**
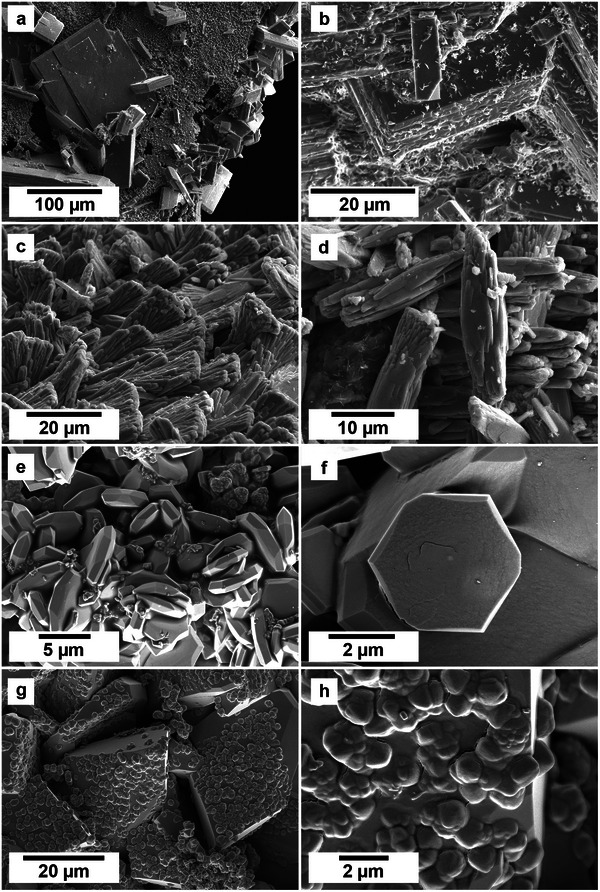
Secondary electron SEM images showing the series of replacement REE‐bearing minerals observed on the surface of the host carbonate grains. a) Thin, platy lanthanite crystals on calcite at 21 °C after 12 weeks in PAAS solution. b) Detail of lanthanite with small (<1 µm) secondary kozoite crystals on aragonite at 21 °C after 8 weeks in PAAS solution. c) Spherulitic kozoite on aragonite at 80 °C after 1 week in PAAS solution. d) Detail of kozoite on aragonite at 80 °C after 1 week in non‐PAAS solution. e) Hydroxylbastnäsite on aragonite at 210 °C after 24 h in non‐PAAS solution. f) Detail of hydroxylbastnäsite on dolomite at 210 °C after 48 h in non‐PAAS solution. g) Secondary replacement of hydroxylbastnäsite by cerianite on calcite at 210 °C after 24 h in non‐PAAS solution. h) Detail of the early stages of nanocerianite formation on calcite at 210 °C after 24 h in non‐PAAS solution.

SEM imaging revealed the formation of precipitates on the surface of the calcite, dolomite and aragonite grains. The extent to which these newly formed precipitates covered and replaced the host carbonate grain was found to be time and temperature dependent. The type of carbonate host as well as the REE concentration in solution also played a key role in the rate of replacement. For example, at ambient temperature, and in the equal concentration solutions, only the aragonite grains were seen to be fully covered by large (50–100 µm) platy lanthanite crystals, while the dolomite and calcite grains experienced only minor replacement (<1%) and few visible small (<10 µm) elongate lanthanite crystals (**Tables** **1**, **2**–**3**). Comparatively, in the PAAS solution, both aragonite and calcite were both fully encased in a crust of lanthanite, while the dolomite still showed some surface area without any replacement, albeit with dissolution textures. At 50 and 80 °C, all three hosts were completely encapsulated by the REE carbonate kozoite (**Figure** **3**). Morphologies of the newly formed crystals consisted of small, elongate prisms of ≈10 µm were found on all three hosts along with spherulitic aggregates in the aragonite experiments in both the equal concentration solution (after 8 weeks) and the PAAS solution (after 24 h). In the higher temperature experiments (165–210 °C) crystal morphologies consisted of triangular prisms of hydroxy‐bastnäsite along with small octahedron of cerianite (**Figure** **4**).

**Table 1:** Experimental conditions, identities and morphologies of the solid rare earth carbonate and oxide phases formed during the interaction of dolomite with multi‐component (La, Ce, Pr, Nd, and Dy), REE‐bearing aqueous solutions (non‐PAAS, and PAAS). *kozoite (koz), hydroxylbastnäsite (HB), cerianite (Cer).

DOLOMITE							
NON‐PAAS					PAAS		
T [°C]	Time [days]	% Phase consumed	Phase formed	Morphology	% Phase consumed	Phase formed	Morphology
21	14	0			6	Lanthanite	Thin platy crystals
	28	0			1		
	42	0			3		
	56	0			1		
	84	0			4		
50	1	0			0		
	3	<1	Kozoite		3	Kozoite	small prisms, elongated prisms
	7	6		small prisms, elongated prisms	5		
	21	7			19		
	28	14			11		
	42	25			20		
	56	19			44		
80	1	0			3	Kozoite	small prisms, elongated prisms
	3	7	Kozoite	small prisms, elongated prisms	9		
	7	14			24		
	21	17			24		
	28	19			15		
	42	48			20		
	56	18			36		
165	1	23	Kozoite	Koz: small prisms, elongated prisms Cer: small octahedron HB: triangular prisms	22	12% Koz, 8% HB, 2% Cer	Koz: small prisms, elongated prisms Cer: small octahedron HB: triangular prisms
	2	16	Kozoite		17	6% Koz, 7% HB, 2% Cer	
	3	18	17% Koz, <1% Cer		40	5% Koz, 29% HB, 6% Cer	
	4	28	27% Koz, <1% Cer		31	2% Koz, 25% HB, 4% Cer	
	7	22	21% Koz, <1% Cer		100	73% HB, 27% Cer	
	14	23	19% Koz, 3% HB, <1% Cer		100	43% HB, 57% Cer	
210	1	24	14% Koz, 5% HB, 5% Cer	Koz: small prisms, elongated prisms Cer: small octahedron HB: triangular prisms	42	25% HB, 17% Cer	Koz: small prisms, elongated prisms Cer: small octahedron HB: triangular prisms
	2	27	16% Koz, 10% HB, <1% Cer		38	21% HB, 17% Cer	
	3	37	13% Koz, 22% HB, 2% Cer		42	24% HB, 18% Cer	
	7	34	33% HB, 1% Cer		41	25% HB, 16% Cer	

**Table 2:** Experimental conditions, identities and morphologies of the solid rare earth carbonate and oxide phases formed during the interaction of aragonite with multi‐component (La, Ce, Pr, ND, and Dy), REE‐bearing aqueous solutions (non‐PAAS, and PAAS). * lanthanite (lan), kozoite (Koz), hydroxylbastnäsite (HB), cerianite (Cer).

ARAGONITE							
NON‐PAAS					PAAS		
T [°C]	Time [days]	% Phase consumed	Phase formed	Morphology	% Phase consumed	Phase formed	Morphology
21	14	0			13	Lanthanite	Thin platy crystals
	28	7	Lanthanite	Thin platy crystals	11		
	42	11			22		
	56	8			15		
	84	18			13		
50	3	12	Kozoite	small prisms, elongated prisms	4	3%Koz,1% Lan	Lan: Thin platy crystals Koz: Small prisms, Elongated prisms
	7	8			12	Koz	
	21	68			11	Koz	
	28				14	Koz	
	42	100			27	25% Koz, 2% Lan	
	56	16			50	48% Koz, 2% Lan	
80	1	11	Kozoite	small prisms, elongated prisms	16	Koz	Koz: small prisms, elongated prisms HB: Triangular prisms
	3	6			9		
	7	9			21		
	21	25			28	23% Koz, 5% HB	
	28	57			23	Koz	
	42	37			42		
	56	50			54		
165	1	13	Koz	Koz: small prisms, elongated prisms Cer: small octahedron HB: triangular prisms	17	14% Koz, 2% HB, 1% Cer	Koz: small prisms, elongated prisms Cer: small octahedron HB: triangular prisms
	2	15	13% Koz, <1% Cer, <1%HB		19	13% Koz, 4% HB, 2% Cer	
	3	18	17% Koz, <1% Cer		29	11% Koz, 15%HB, 3% Cer	
	4	25	24% Koz, 1% Cer		34	12% Koz, 19% HB, 3% Cer	
	7	39	37% Koz, 2% Cer		60	2% Koz, 45% HB, 13% Cer	
	14	25	23% Koz, <1% Cer, <1% HB				
210	1	9	7% Koz, 1% Cer		43	3% Koz, 23% HB, 17% Cer	
	2	11	6% Koz, 4% HB, 1% Cer		45	29% Hb, 16% Cer	
	3	31	12% Koz, 16% HB, 3% Cer		48	30% HB, 18% Cer	
	7	38	8% Koz, 28% HB, 2% Cer		64	49% HB, 15% Cer	

**Table 3:** Experimental conditions, identities and morphologies of the solid rare earth carbonate and oxide phases formed during the interaction of calcite with multi‐component (La, Ce, Pr, ND, and Dy), REE‐bearing aqueous solutions (non‐PAAS, and PAAS). *lanthanite (Lan), kozoite (Koz), hydroxylbastnäsite (HB), cerianite (Cer).

CALCITE							
NON‐PAAS					PAAS		
T [°C]	Time [days]	% Phase consumed	Phase formed	Morphology	% Phase consumed	Phase formed	Morphology
21	14	0			6	Lan	Thin platy crystals
	28	0			0		
	42	0			0		
	56	0			0		
	84	0			10		
50	1	0			0		
	3	0			0		
	7	3	Kozoite	small prisms, elongated prisms	1	Kozoite	small prisms, elongated prisms
	21	7			7		
	28	7			11		
	42	12			37		
	56	14			29		
80	1	0			<1	Koz	small prisms, elongated prisms
	3	7	Kozoite	small prisms, elongated prisms	11	6% Koz, 5% Lan	
	7	10					
	21	36			40	Koz	
	28	67					
	42	76			17	Koz	
	56	53			41	Koz	
165	1	41	Koz	Koz: small prisms, elongated prisms Cer: small octahedra HB: triangular prisms	28	25% Koz, 2% HB, <1% Cer	Koz: small prisms, elongated prisms Cer: small octahedra HB: triangular prisms
	2	25	Koz		32	30% Koz, <1% HB, <1% Cer	
	3	49	Koz		35	31% Koz, 3% HB, 1% Cer	
	4	54	52% Koz, <1% Cer, <1% HB		29	25% Koz, 3% HB, 1% Cer	
	7	38	35% Koz, 1% Cer, 2% HB		37	19% Koz, 13% HB, 5% Cer	
	14	38	35% Koz, <1% Cer, 2% HB				
210	1	20	16% Koz, 4% Cer	Koz: small prisms, elongated prisms Cer: small octahedra HB: triangular prisms	72	37% HB, 35% Cer	Koz: small, elongated prisms Cer: small octahedra HB: triangular prisms
	2	13	11% Koz, 1% HB, <1% Cer		100	54% HB, 46% Cer	
	3	17	14% Koz, 3% HB		95	58% HB, 37% Cer	
	7	100	Cerianite		80	47% HB, 33% Cer	

**Figure 5:**
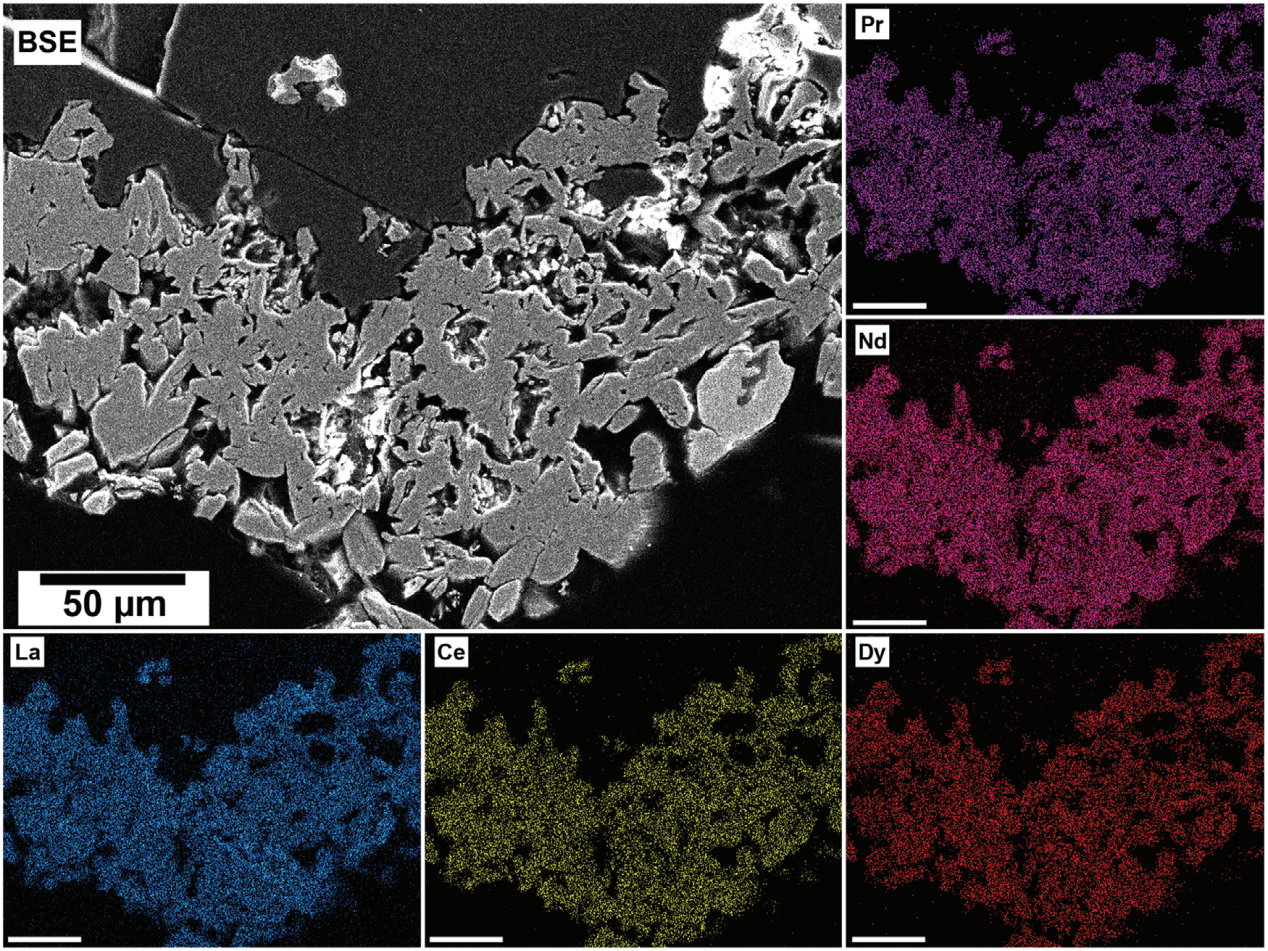
SEM‐BSE image of calcite grain at 165 °C after 48 h in non‐PAAS solution and EDS maps of La (blue), Ce (yellow), Pr (purple), Nd (pink), and Dy (red) showing the homogenous distribution of the REE.

**Figure 6:**
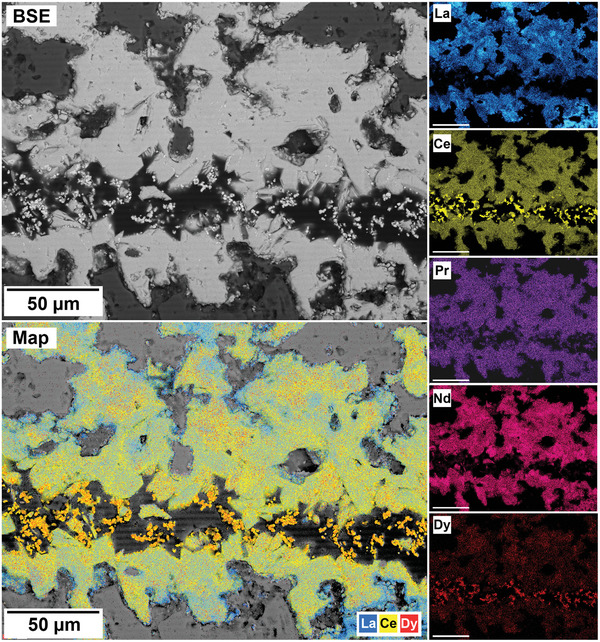
SEM‐BSE image and EDS elemental maps of an aragonite grain at 165 °C after 24 h in PAAS solution showing the heterogenous distribution of the REE and the association of cerianite (yellow) and Dy (red).

**Figure 7:**
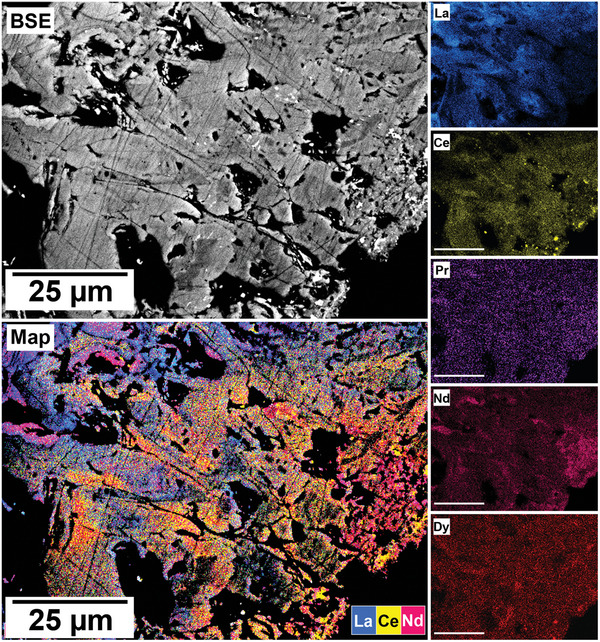
SEM‐BSE image and EDS elemental maps of a calcite grain at 165 °C after 96 h in PAAS solution showing potential zoning in kozoite crystal, with lighter REE concentrated in the centre and heavier REE in the outer edges of the crystals.

**Figure 8:**
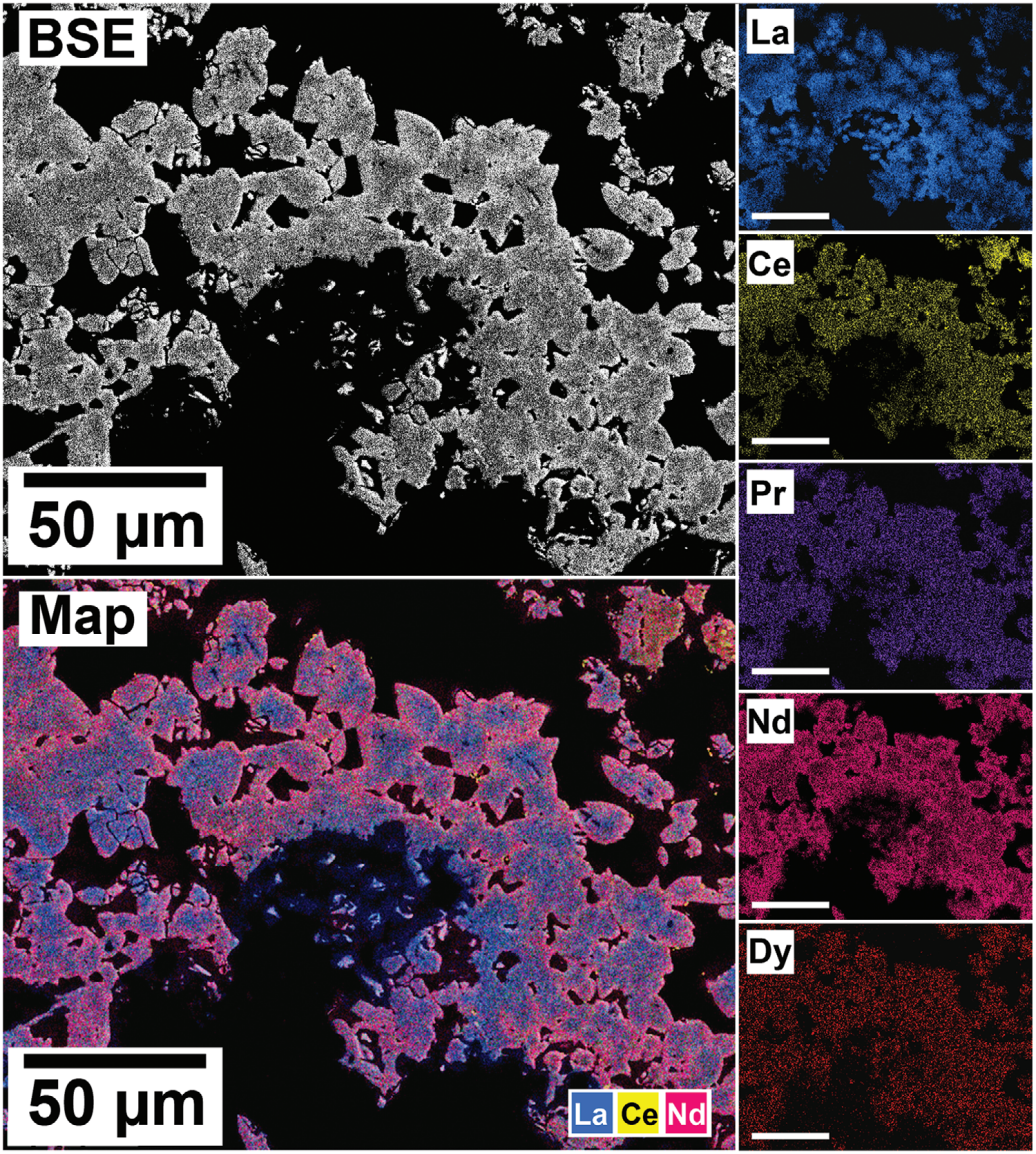
SEM‐BSE image and EDS elemental maps of an aragonite grain at 165 °C after 72 h in PAAS solution showing potential zoning in kozoite crystal, with lighter REE concentrated in the centre and heavier REE in the outer edges of the crystals.

**Figure 9:**
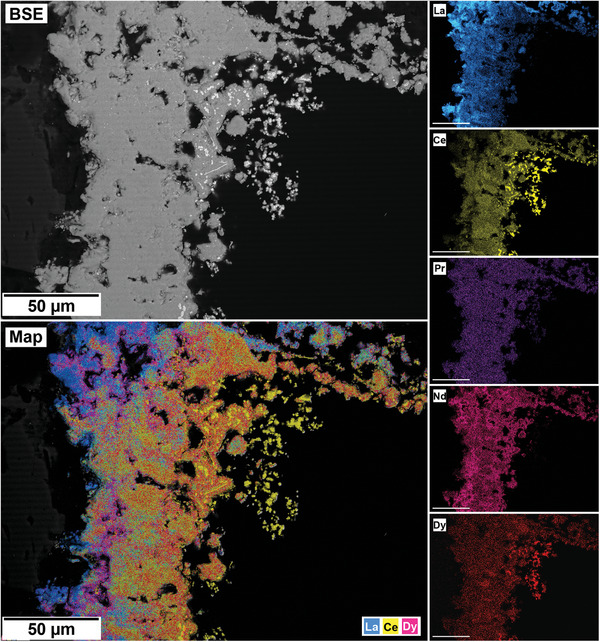
SEM‐BSE image and EDS elemental maps showing the compositional gradation of REE in the replacement corona surrounding the aragonite host at 165 °C after 96 h in PAAS solution, with lighter REE accumulated toward the inner rims and heavier REE in the outer rim.

The polished epoxy resin pucks were subjected to energy dispersive spectroscopy (EDS). By mapping the cross section of the grains, differences in the spatial distribution of the REEs as they replaced the carbonate host grains were recorded. The replacement trends were found to be dependent on the initial concentration of REE in solution, with a more homogenous distribution in the equal concentration solution (**Figure** **5**), and a more heterogenous distribution in the PAAS solution (**Figure** **6**). The SEM‐EDS analyses of the REE within the overgrowth revealed intriguing differences between the PAAS and non‐PAAS samples, showing variations in the composition of both the complete overgrowth and localized differences in individual crystals. Although this trend is consistent across all experiments, both SEM‐EDS and SEM‐BSE analyses showed more pronounced chemical variations in the PAAS samples compared to the non‐PAAS samples. In all instances, these compositional variations indicate two main trends: the first involves potential zoning, with lighter elements concentrated in the centre and heavier elements in the rims of the crystal (**Figures** **7** and **8**; Figure S2, Supporting Information), similar to the observations of Miyawaki et al.^[^ ^11^ ^]^ Similarly, the second trend comprises a general compositional gradation in the replacement corona surrounding the Ca–Mg carbonate host, also featuring lighter REE elements in the inner rim and heavier elements in the outer rim (**Figure** **9**; Figure S3, Supporting Information). Despite the occurrences of these chemical variations, there are limitations in our quantitative determinations which arise from the small size of individual kozoite/bastnäsite crystals (<20 µm) within the replacement corona, impeding detailed scrutiny, coupled with the subtle nature of compositional REE variations. These factors constrain the ability to carry out a precise quantification of these compositional variations.

**Figure 10:**
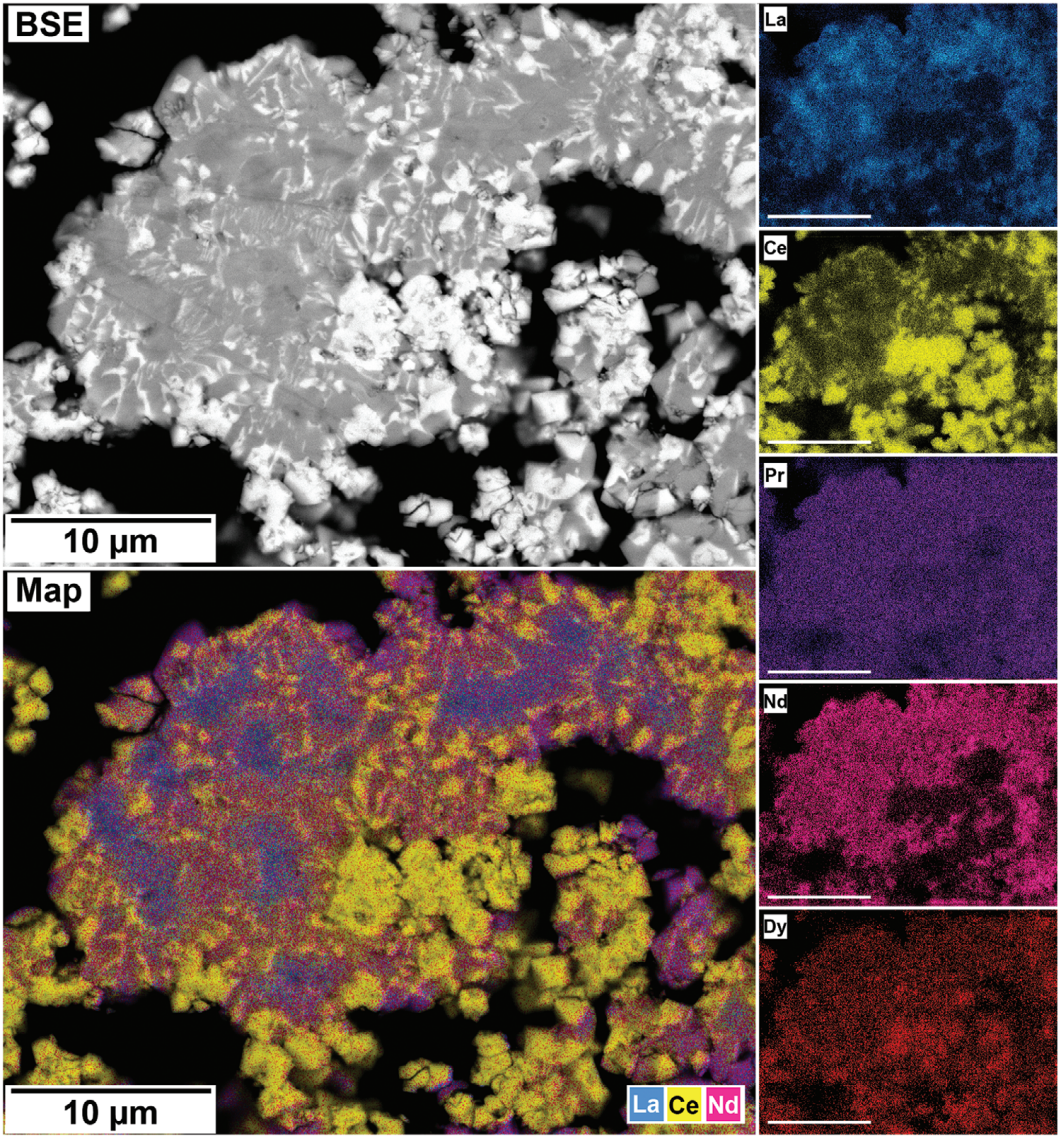
SEM‐BSE image and EDS elemental maps of an aragonite grain at 210 °C after 24 h in PAAS solution. Areas of La‐rich areas surrounded by vermicular intergrowths of Nd and Ce radiating outwards form the symplectic textures.

**Figure 11:**
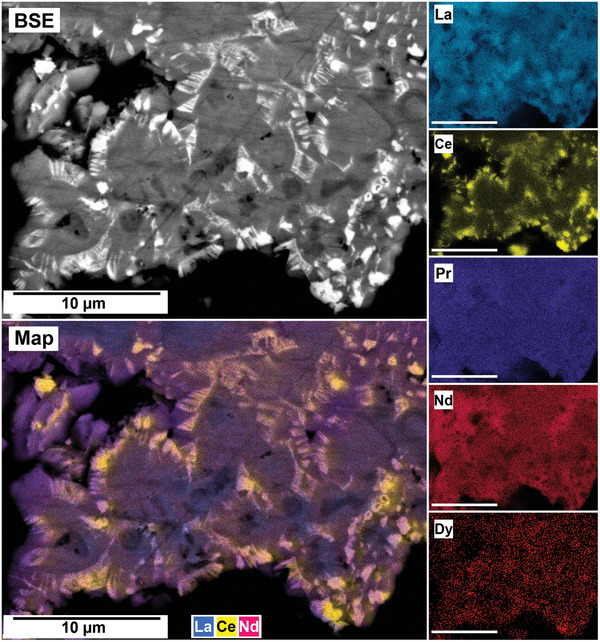
SEM‐BSE image and EDS elemental maps of an aragonite grain at 210 °C after 24 h in PAAS solution showing how the location of cerianite defines the boundaries between pre‐existing spindle‐shaped kozoite crystals.

Another feature observed in the cross‐section EDS maps was symplectic textures, particularly in the PAAS samples subjected to the highest temperature of 210 °C. In the aragonite grains, we can see La, Pr‐rich areas surrounded by vermicular intergrowths of Nd and Ce, radiating outwards (**Figure** **10**), further supporting the idea that the heterogenous distribution of REEs is dependent on the REE initial concentration in solution. In addition to the symplectic textures, we also observed areas with a significant accumulation of cerianite, seemingly having fully replaced the rare earth carbonate minerals, with Dy being associated with Ce. (Figures 10 and **11**).

## 3 Discussion

**Figure 12:**
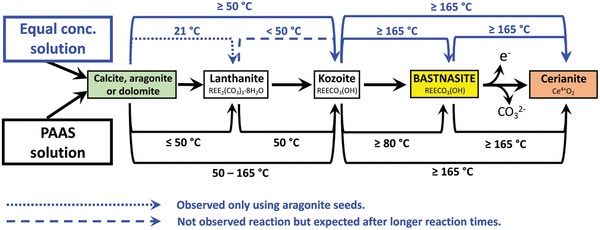
Reaction pathways during the replacement of Ca(‐Mg) carbonates by rare earth carbonates and cerianite. Continuous lines correspond to observed reactions, dotted lines to observed reactions only using aragonite seeds, while dashed lines correspond to expected reactions that were not observed in our experiments. Each line shows the temperature at which the reaction takes place.

The interaction of the Ca–Mg carbonates with the REE‐bearing solutions resulted in the initial dissolution of the host grain, releasing Ca^2+^ and CO_3_2^−^ (and Mg^2+^ from dolomite) into the aqueous solution. This dissolution of the host Ca(‐Mg) carbonates rapidly increased the supersaturation of the solution, especially at the carbonate surface/solution interface, triggering the surface precipitation of REE‐bearing carbonates, with some grains being fully replaced from the periphery inwards. Also, at the highest temperatures (210 °C), a secondary replacement occurred, with the REE‐carbonate minerals being partially or totally replaced by cerianite. The reaction sequence (lanthanite → kozoite → hydroxylbastnäsite → cerianite) was time‐ and temperature‐dependent, with some of the intermediate, metastable phases not forming at higher temperatures (≥ 165 °C) (**Figure** **12**). The rate of crystallization and composition of the main REE‐bearing phases as well the observed textures (Figures 5, 6, 7, 8, 9, 10, 11) were controlled by the solubilities and dissolution rates of the host grains and the REE ratio in solution, combined with the evolving fluid composition during the gradual replacement process. The influence of temperature is also a key factor, as it affects the dehydration of specific REE^3+^ before being incorporated into the growing crystals as well as the oxidation of Ce^3+^ to Ce^4+^ in solution.

### 3.1 Solubility, Dissolution Rate and Chemical Textures

In all of our experiments we observed a centripetal replacement of the host mineral, as they were replaced from the periphery inwards by the newly formed REE carbonates. This is a consequence of the lower solubilities of REE carbonates compared to the host minerals at the specific pressure‐temperature conditions studied.^[^ ^12^, ^13^, ^14^ ^]^ For example, the solubility products for pure Nd‐hydroxylbastnäsite, La‐hydroxylbastnäsite, and Nd‐Kozoite have been calculated to be log(K_sp_) = −23.8 ± 0.1, log(K_sp_) = −24.1 ± 0.3, and log(K_sp_) = −22.3 ± 0.2, respectively,^[^ ^13^ ^]^ while the solubility products of calcite, log(K_sp_) = −8.480 ± 0.020, aragonite, log(K_sp_) = −8.336 ± 0.020^[^ ^15^ ^]^ and dolomite, log(K_sp_) = −18.14^[^ ^16^ ^]^ are much higher. Determining the solubility of solid phases with intermediate compositions is not feasible due to the absence of relevant data. Existing literature suggests that lighter REE carbonates are more soluble than their heavier counterparts.^[^ ^12^ ^]^ However, conflicting trends and discrepancies have been reported by other researchers,^[^ ^13^, ^14^ ^]^ highlighting the complexity of this system, with some reported variations potentially arising from the occurrence of different crystalline (nano)phases during synthesis, each possessing different solubility products and different kinetics of crystallization.^[^ ^17^, ^18^ ^]^ Also, measuring the solubility products at elevated temperatures is challenging because of insufficient availability of reliable date pertaining to the stability of diverse aqueous complexes formed by REEs with carbonate and hydroxyl ions.^[^ ^13^ ^]^

While these large differences in solubility influence the polymorph selection and its chemistry, it is the dissolution rate of the host mineral that determines the rate at which the new phase can precipitate. For example, the dissolution rates of calcite, aragonite and dolomite at 25 °C have been calculated to be log R_cal_ = −8.8 mol^−2^ s^−1^, R_arg_ = −9.2 mol^−2^ s^−1^ and log R_dol_ = −10.1 mol^−2^ s^−1 [^ ^19^ ^]^ meaning that, e.g., aragonite will dissolve almost an order of magnitude faster than dolomite at the initial pH of 5.1. This is particularly evident in our ambient and 50 °C experiments, with replacement of aragonite commencing after 3 days compared to the replacement of calcite and dolomite initiating after 7 and 21 days respectively. The higher dissolution rates of aragonite and calcite mean that carbonate ions are released faster into the aqueous solution compared to the dolomite experiments, resulting in higher supersaturation levels needed to initiate the nucleation of the REE carbonates. Using calcite as an example, PHREEQC calculations show that even when the saturation index for calcite is −5, the concentration of Ca^2+^ and CO_3_2^−^ released into the aqueous solution is high enough to allow the supersaturation with respect to REE carbonate minerals. Therefore, at ambient temperature, even small amounts of the host mineral dissolution would promote the precipitation and growth of REE carbonates (e.g., lanthanite in our ambient temperature experiments). Another factor to consider is that we did not stir our experiments, meaning that our grains remained static inside the reactors. As a consequence, the concentrations of Ca^2+^, Mg^2+^, and CO_3_^2−^ at the interface between the host mineral and the aqueous solution should have been even higher, resulting in higher supersaturation levels with respect to REE carbonates. This means that dissolving just a few monolayers of the host grain would sufficiently promote the nucleation and surface crystallization of the REE carbonates. This process, in turn, would lower the concentration of CO_3_^2−^ in the solution, further enhancing the dissolution of the host grain.

Additionally, it is important to emphasize the role of ionic potentials of the REE^3+^ cations in this process.^[^ ^20^ ^]^ Lighter trivalent ions require lower energy for dehydration from solution compared to heavier ones,^[^ ^17^, ^18^ ^]^ kinetically favoring the formation of La‐rich crystals in the initial stages of the replacement reaction. As the lighter elements are gradually consumed, the solution becomes enriched in heavier ions, resulting in the development of crystals characterized by heavier‐REE rims.

Although our SEM‐BSE‐EDS analyses of REE carbonates (Figures 5, 6, 7, 8, 9; Figures S2 and S3, Supporting Information) would suggest lighter REE carbonates exhibiting lower solubility compared to their heavier counterparts, it is crucial to emphasize that these replacement reactions present a multi‐variable challenge. The chemical textures observed in kozoite may be subject to local reorganization during its transformation to secondary bastnäsite. This gradual process observed in our experiments (Tables 1, 2, 3, Figure 2), is known to occur via dissolution‐recrystallization^[^ ^17^, ^18^, ^21^, ^22^, ^23^ ^]^ and introduces an additional layer of complexity, potentially influencing the complex chemical variations occurring in the replacement rim at the micro‐ and nanoscale. Overall, the intricate interplay of crystal structures, variable solid‐solution compositions and solubilities, ionic potentials, temperature, evolving solution composition, available reactive surface, as well as temperature‐dependent dissolution‐recrystallization processes, collectively contribute to the observed chemical variability within the single crystals and in the replacement ring. Quantifying the preferential incorporation of REEs in our samples is also highly complicated due to the small size and random orientation of the individual kozoite or bastnäsite crystals within the replacement corona. Therefore, a more comprehensive examination would require the production of larger or isolated single crystals, a task that exceeds the scope of the current study.

#### 3.1.1 The Effect of Molar Volume

With the exception of two of our experiments (e.g., dolomite, PAAS solution at 165 °C, and calcite, non‐PAAS solution at 210 °C), none of the experiments resulted in the complete replacement of the host grain. This can be explained by difference in molar volume between the minerals in the host grain and the overgrowth. If the newly forming REE carbonate overgrowth has a larger molar volume compared to the host mineral, it will begin to encapsulate the host. As the host is being covered, its dissolution rate will decrease, triggering a decrease in REE carbonate precipitation as there are less CO_3_^2−^ ions entering the aqueous solution. When this overgrowth is large and dense enough, and completely isolates the host grain from the aqueous solution, the dissolution of the host stops and a ’partial equilibrium’ with respect to the REE carbonates is reached.^[^ ^24^ ^]^ We can use the reaction of calcite (molar volume of 36.93 cm^3^ mol^−1^) being replaced by lanthanite (molar volume 216.70 cm^3^ mol^−1^) as an example:

(1) $$3\mathrm{CaC}O_{3}+\mathrm{RE}E^{3+}+8H_{2}O\to \mathrm{RE}E_{2}{\left(CO_{3}\right)}_{3}\cdot 8H_{2}O+3Ca^{2+}$$

The difference between the molar volumes of the reagents and the products is so great that the lanthanite replacement can fully encapsulate the host. In our ambient temperature experiments, the maximum consumption of the host phase was only 18%, even after a duration of 84 days (Tables 1, 2, 3). Comparatively, we can look at the replacement of calcite by kozoite or bastnäsite (molar volumes 45.48 and 44.91 cm^3^ mol^−1^ respectively):

(2) $$\mathrm{CaC}O_{3}+\mathrm{RE}E^{3+}+H_{2}O\to \mathrm{REEC}O_{3}\mathrm{OH}+Ca^{2+}+H^{+}$$

Here, the difference in molar volumes also results in the encapsulation of the host, however, the difference is not substantial compared to the difference between lanthanite and calcite. This very small difference in molar volume, combined with the morphology of kozoite and bastnäsite consisting of many small micrometric crystals, allows for the development of microporosity during the replacement process. This causes some fluid to remain in contact with the host grain, enabling its dissolution and the potential full replacement of the host grain, as seen in our high temperature (>165 °C) experiments.

*Secondary replacement of the REE carbonates with cerianite and formation of symplectic textures*

In all of our experiments above 165 °C we observed a secondary replacement of kozoite and hydroxylbastnäsite by cerianite (Figure 10). The onset of cerianite formation is a consequence of the oxidation of Ce^3+^ to Ce^4+^, with several factors promoting this transition. First, Ce^4+^ has the electron structure of a noble gas, making it more stable than Ce^3+^, and the higher temperatures provide the energy needed to facilitate the loss of an electron. Also, as our systems progressed toward equilibrium, the pH would increase from ≈5 to 8.1–8.2. The higher concentration of OH^−^ ions allows the formation of Ce‐hydroxo‐complexes which facilitate the Ce^3+^ to Ce^4+^ oxidation process,^[^ ^21^, ^25^ ^]^ especially at the surface‐solution interface of the REE or Ca‐Mg‐carbonate minerals.^[^ ^21^, ^25^ ^]^ As Ce^4+^ is incompatible within the carbonate structure, it combines with oxygen, forming the oxide cerianite (CeO_2_), which is the most insoluble and the most thermodynamically stable phase in our system. Our experimental setup (sealed hydrothermal reactors containing a limited atmosphere, under conditions of autogenous pressure) inherently creates an oxidising environment favoring the oxidation of Ce^3+^ to Ce^4+^ reaction. Had we conducted the experiments using anoxic water and a pure N_2_ atmosphere, the presence of carbonates like calcite and dolomite would have also buffered the pH of the aqueous solution, increasing the stability of the HCO_3_^−^ ions relative to CO_2_, but these changes would have a negligible influence on the redox state. This means that the formation of cerianite would likely not have occurred and Ce would have been incorporated to the newly formed carbonates as Ce^3+^ along with the other REEs in the system.

The SEM‐EDS images and maps (Figures 10 and 11) show a very specific intergrowth of the REE carbonates and cerianite at high temperatures (>165 °C), a textural feature known as a symplectite. These textural features are common in metamorphic rocks that have undergone high pressure‐temperature conditions^[^ ^26^ ^]^ and have also been found in REE‐bearing deposits.^[^ ^27^ ^]^ The exact mechanisms behind the formation of symplectites can vary depending on the specific mineral assemblages and geological conditions. However, we propose that in our experiments this texture results from a fluid‐mediated mineral replacement, driven by dissolution‐precipitation mechanisms and intra‐mineral diffusion processes.^[^ ^28^ ^]^ In all of our experiments, all of the REE carbonates formed prior to cerianite and the latter always crystallized exclusively in association with the surface of the pre‐existing REE carbonates, indicating that this process takes place at the solution‐surface interface, in agreement with Szucs et al (2023). As cerianite is more insoluble (log(K_sp_) = −59.3 ± 0.3;^[^ ^29^ ^]^ compared to kozoite (log(K_sp_) = −22.3 ± 0.2) or bastnäsite (log(K_sp_) = −23.8 ± 0.1) or the host Ca–Mg carbonates, it can replace any carbonate crystals in our system until the Ce^4+^ is depleted from the aqueous solution. This replacement is also enhanced by the smaller molar volume of cerianite (23.86 cm^3^ mol^−1^) compared to kozoite (45.59 cm^3^ mol^−1^) or bastnäsite (44.91 cm^3^ mol^−1^) which increases the porosity of the parent phase.^[^ ^30^ ^]^ The kozoite / bastnäsite replacement by cerianite can be expressed by the following reaction:

(3) $$\begin{array}{ccc} & & \left(Ce_{x},\mathrm{RE}E_{1-x}\right)CO_{3}\mathrm{OH}+Ce^{4+}\to \mathrm{xC}e^{4+}O_{2(s)}+\mathrm{RE}{E^{3+}}_{1-x}\\ & & \;+\;CO_{2(\mathrm{aq})}+{H^{+}}_{\left(\mathrm{aq}\right)}+e^{-} \end{array}$$

As the molar volume of cerianite is the smallest of all phases present in our system, when this oxide replaces any REE carbonate, or the host, it creates porosity. This allows fluid access to the pre‐existing REE carbonates, and the continuation of the replacement reaction, releasing previously immobilized REEs from the carbonate minerals back into the solution. As some of the REE have similar ionic radii to Ce^4+^, in particular Dy^3+^, and to a slightly lesser extent, Nd^3+^, they can be incorporated into cerianite as impurities. If there is enough Ce in the system, cerianite has the potential to fully replace all of the other mineral phases, with the remaining incompatible REE^3+^ ending up in the aqueous solution.

Concomitantly, any Ce^3+^ located in the non‐replaced kozoite or bastnäsite structures will also tend to oxidize to Ce^4+^. Assuming an REE‐O coordination of 9 in kozoite and bastnäsite, the Ce^3+^ to Ce^4+^ oxidation is also translated into a decrease in the ionic radius from 1.33 to 1.16 Å,^[^ ^20^ ^]^ making Ce^4+^ incompatible with these REE carbonates. Therefore, the oxidation process would trigger a solid‐state phase transformation from REE‐carbonates to cerianite at the local scale. This process would involve the migration of Ce^4+^ ions through the existing REE carbonate structure, leading to their accumulation at the nanoscale, triggering the nucleation of nm‐sized cerianite. Our EDS maps (Figures 10 and 11) reveal a depletion of Ce in the REE carbonate phases and the accumulation of this element as a discrete phase (cerianite), while the rest of the REEs tend to concentrate in the carbonate crystals.

Both our SEM‐BSE images and EDS maps (Figures 10 and 11) show that the location of cerianite defines the boundaries between pre‐existing spindle‐shaped kozoite crystals. This indicates that the formation of these symplectic textures has been favored by the boundaries of the original polycrystalline material, as it is easier for ions to migrate through crystal imperfections. Therefore, the local boundaries between the parent kozoite single crystals provide the ideal starting point to initiate the formation of the symplectic texture, in turn creating the visible “ghosts” textures of the pre‐existing kozoite.

## 4 Conclusion

The reaction between each of the carbonate host grains, dolomite, aragonite and calcite, with multi‐component REE‐bearing (La, Ce, Pr, Nd, and Dy) aqueous solutions resulted in the replacement of the carbonate host and the formation of secondary REE‐bearing minerals. The reaction followed the multi‐step crystallization sequence: lanthanite → kozoite → hydroxylbastnäsite → cerianite. The REEs were preferentially incorporated into the growing REE‐minerals, likely because of the combination of several factors including the solubility and dissolution rate of the carbonate host compared to the REE minerals, time, temperature, ionic radius and ionic potential of the REE as well as its concentration in solution. At higher temperatures (>165 °C) a secondary replacement of kozoite and bastnäsite by the rare earth oxide, cerianite, was observed. Differences in molar volumes between cerianite and kozoite/bastnäsite generated porosity in the newly formed mineral and allowed for solution‐mediated mineral replacements, producing symplectic textures. Our study sheds light on the uptake of multiple REEs by various carbonate minerals and provides insight into the potential zoning of REEs in rare earth minerals. A broader knowledge of REE zoning will have potential applications in rare earth separation techniques, beneficiation, and recycling.

## 5 Experimental Section

The interaction of calcite, dolomite and aragonite with multi‐component REE (La, Ce, Pr, Nd, and Dy) aqueous solutions was investigated at ambient and hydrothermal conditions (21–210 °C). Calcite (Iceland spar), aragonite (Sputnik variety) and light‐pink dolomite (Morocco) crystals were crushed in a ceramic mortar and sieved to extract 0.5–1.0 mm clasts. Five single REE bearing solutions were prepared, (La, Ce, Pr, Nd, and Dy), with a REE concentration of 50 mm. These solutions were produced via the dissolution of single rare earth element nitrate salts, REE(NO_3_)_3_∙6H_2_O (Sigma–Aldrich, 99.9% purity), in de‐ionized water (18.18 MΩ cm). These five specific REE were chosen as they were representative of both the light (La, Ce, Pe, and Nd) and heavy REE (Dy). This suit of REE from La to Dy also represents 72% of the ionic radii of the lanthanides as well as being some of the most abundant REE in the earth's crust.^[^ ^31^ ^]^ In order to understand the effect of ionic radii of the five REEs and to what extent these ions were taken up by the carbonate host, two different sets of experiments were conducted, one with equal concentrations of the five REEs and one with the concentrations of the five REEs normalized to the commonly used Post Archean Australian Shale standard (PAAS)^[^ ^32^ ^]^ to mimic the rare earth element concentrations found in continental crust and natural geo fluids. For the equal concentration experiments 4 mL of each of the five REEs were used to give a total solution volume of 20 mL for each experiment. For the PAAS experiments, a 1 L bulk solution was made and 20 mL of this was used for each experiment.

For each experiment, 0.1 g of either calcite, dolomite, or aragonite grains were added to 20 mL of each of the 50 mm REE‐bearing solutions and placed in 25 mL Teflon‐lined and capped stainless‐steel autoclaves (for the 80, 165, and 210 °C experiments). For the lower temperatures (21 and 50 °C), narrow‐mouth Nalgene polypropylene PPCO bottles (Thermo Fisher Scientific) were used. The reactors were then placed in a pre‐heated oven at 50, 80, 165, and 210 °C. The room temperature experiments were kept undisturbed in a laboratory cupboard. Solid samples were then extracted using a sterilized metal spatula at increasing time intervals from 24 h to 8 weeks. The samples were placed in plastic Eppendorf tubes and dried in a 30 °C oven, with the exception of the 21 °C samples, which were dried at room temperature. A summary of the experimental protocol is presented in Figure S1 (Supporting Information).

In order to identify and quantify the formation of crystalline solids present in our samples, several grains were selected from each experiment ensuring that each time and temperature variable was represented. The selected grains were ground to a consistently fine powder using an agate pestle and mortar. The powders were analyzed, and minerals characterized with powder X‐ray diffraction (XRD). Conventional powder XRD patterns were collected using a Siemens/Bruker D5000 powder X‐ray diffractometer (Cu Kα radiation, 0.01° step^‐1^ from 5 to 60° 2*θ* at 0.2° min^−1^, 4.5 h scans per samples). Identification of crystalline phases was carried out with the Diffract Suite EVA software from Bruker in combination with the Powder Data File (PDF‐4, the International Centre for Diffraction Data).^[^ ^33^ ^]^ Pattern‐matching refinement and quantification of crystalline phases were carried out with the Rietveld refinement software TOPAS.^[^ ^34^ ^]^ Finally, scanning electron microscopy (SEM) was used to obtain high resolution images to characterize changes in the morphology of the carbonate host grains and identify newly formed phases. Analyses were conducted in the iCRAG laboratory at Trinity College Dublin using a TIGER S8000 FEG‐SEM operating under high vacuum conditions and equipped with two Oxford X‐Max 170 mm^2^ EDS detectors and an X4 pulse processor running the Oxford Aztec analysis software.

Two methods of sample preparation were employed, i) standard SEM sample stubs with intact grains mounted to examine surface morphology, and ii) polished epoxy resin pucks that provided a cross section of the grains, allowing for the identification of potential differences in spatial distribution and internal chemical textures. The epoxy mounts (2.5 cm wide) were made using Epoxy resin (Struers Epofix) mixed with the accompanying hardener and then polished using a three‐step process (Struers Diapro, 6 and 1 µm) to expose the internal cross section of the grains. The pucks were then cleaned in an ultrasonic bath of deionized water to remove any residual polishing fluid and then dried. Both the stubs and the pucks were then coated with either carbon (for elemental mapping and analysis) or gold (for high resolution imaging). Analysis at a working distance of 15 mm was performed using an accelerated voltage of 20 kV, while imaging carried out at a working distance of 5 mm was performed using an accelerating voltage of 10 kV. The images and maps were processed using the AZtec v6.1 X‐ray microanalysis software suit (Oxford Instruments).

## Conflict of Interest

The authors declare no conflict of interest.

## Supporting information

Supporting Information

## Data Availability

The data that support the findings of this study are available from the corresponding author upon reasonable request.
