# Supplementary material for: Chemical Textures on Rare Earth Carbonates: An Experimental Approach to Mimic the Formation of Bastnäsite
Source: Glob Chall. 2024 May 17;8(7):2400074. doi: 10.1002/gch2.202400074 (PMC11237176; doi:10.1002/gch2.202400074)

Global Challenges
Open Access

Supporting Information

for *Global Challenges.*, DOI 10.1002/gch2.202400074

Chemical Textures on Rare Earth Carbonates: An Experimental Approach to Mimic the Formation of Bastnäsite

Melanie Maddin, Remi Rateau, Adrienn Maria Szucs, Luca Terribili, Brendan Hoare, Paul C. Guyett and Juan Diego Rodriguez-Blanco**

Supporting information for the paper:

Chemical textures on rare earth carbonates: an experimental approach to mimic the formation of bastnasite

Melanie Maddin[1*], Remi Rateau[1], Adrienn Maria Szucs[1], Luca Terribili[1], Brendan Hoare[1], Paul C. Guyett[1], Juan Diego Rodriguez-Blanco[1*]

[1]Department of Geology, School of Natural Sciences, Trinity College Dublin. Dublin 2, Ireland.

* maddinm@tcd.ie, J.D.Rodriguez-Blanco@tcd.ie

Figure SI-1. Graphical summary showing the experimental protocol used in this study.

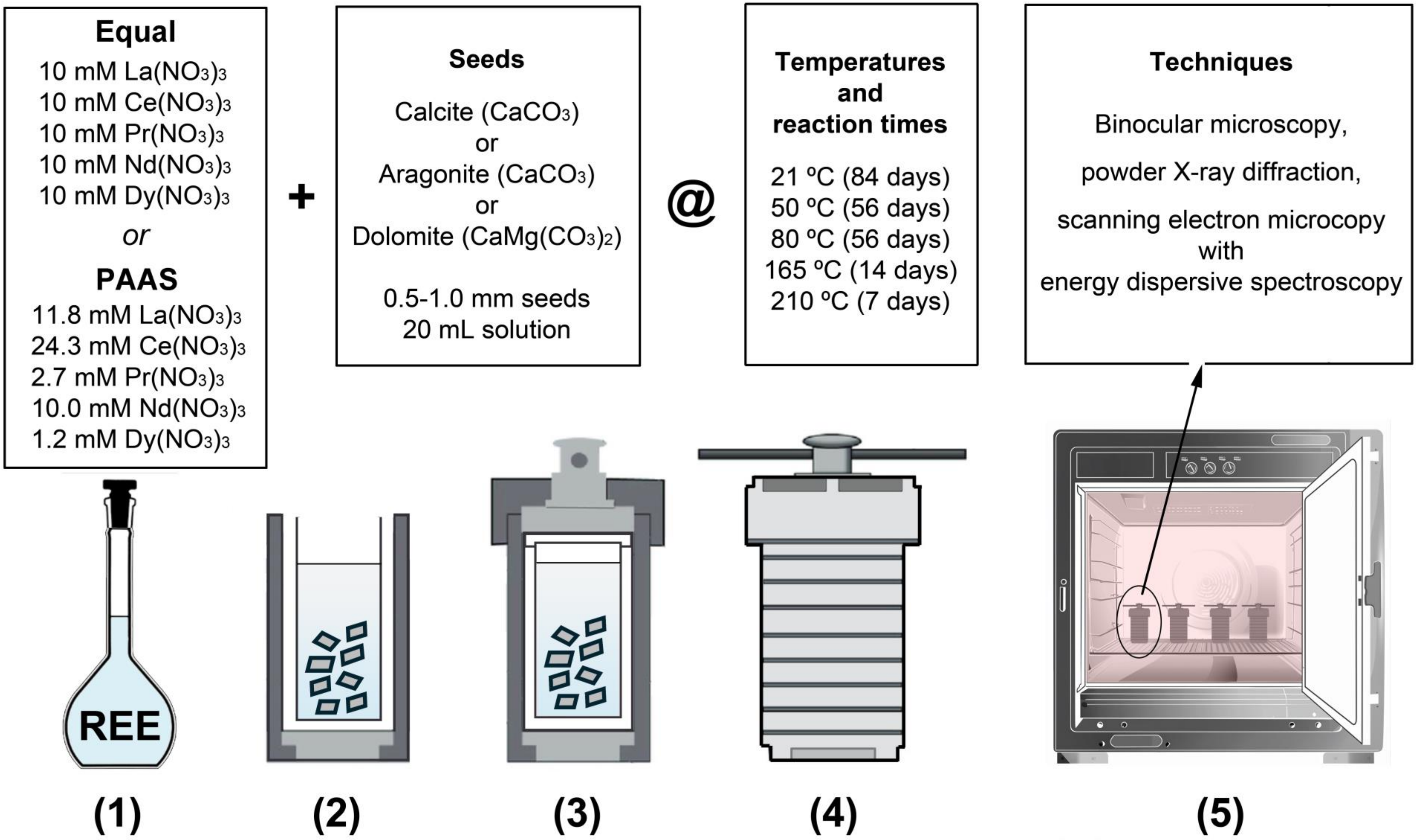

Figure SI-2. SEM-BSE image and EDS elemental maps of an aragonite grain at 165 °C after 72 hrs in PAAS solution showing potential zoning in kozoite crystals, with lighter REE concentrated in the centre and heavier REE in the outer edges of the crystals. Quantitative line analysis (Lines 1, 2 and 3) further supports this trend showing higher concentrations of La in the centre and Nd at the rim of the individual kozoite crystals.

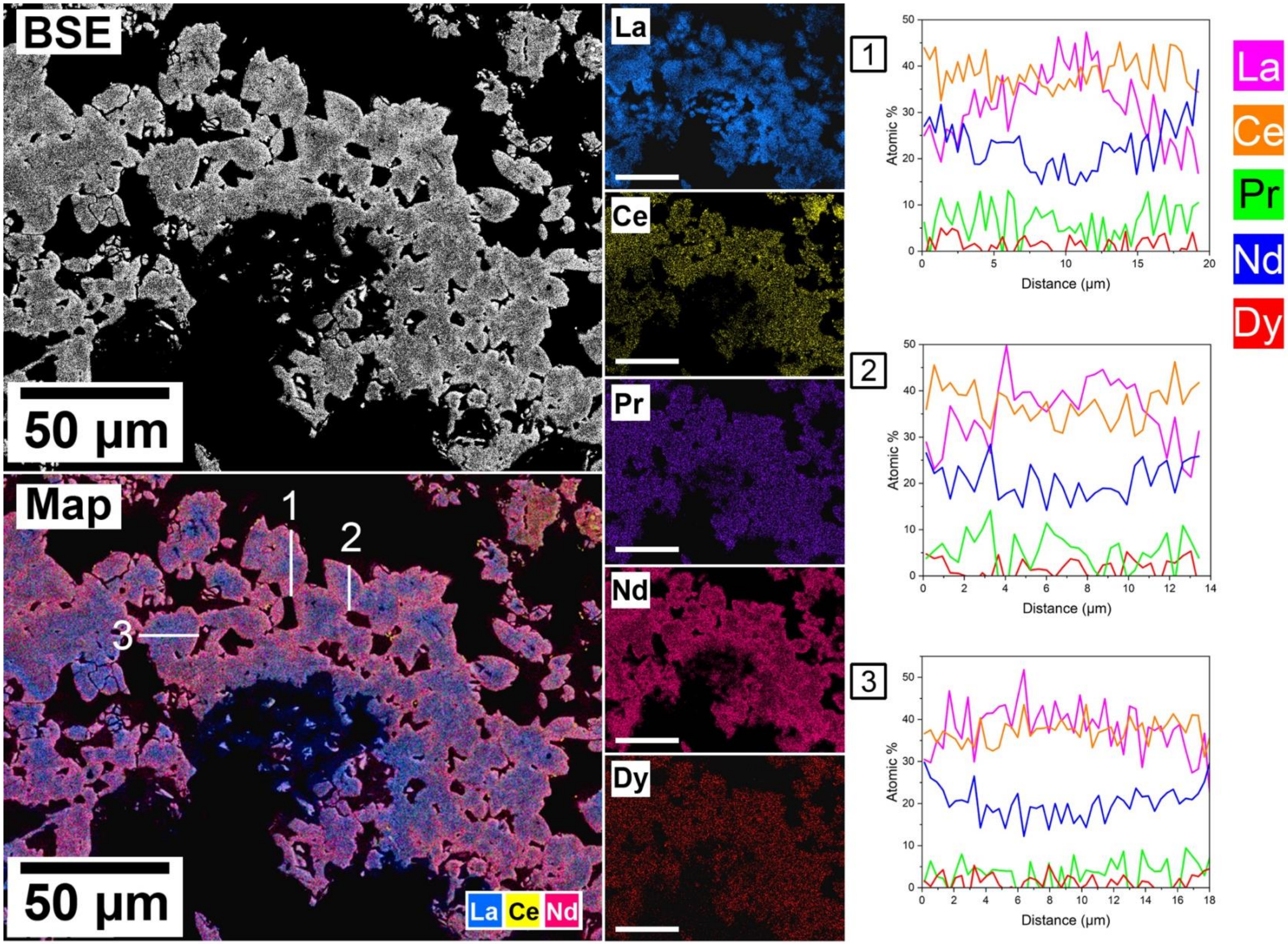

Figure SI-3.. SEM-BSE image and EDS elemental maps showing the compositional gradation of REE in the replacement corona surrounding the aragonite host at 165 °C after 96hrs in PAAS solution, with lighter REE accumulated towards the inner rim and heavier REE in the outer rim. This trend is further supported by quantitative line analysis in lines 1, 2 and 3.

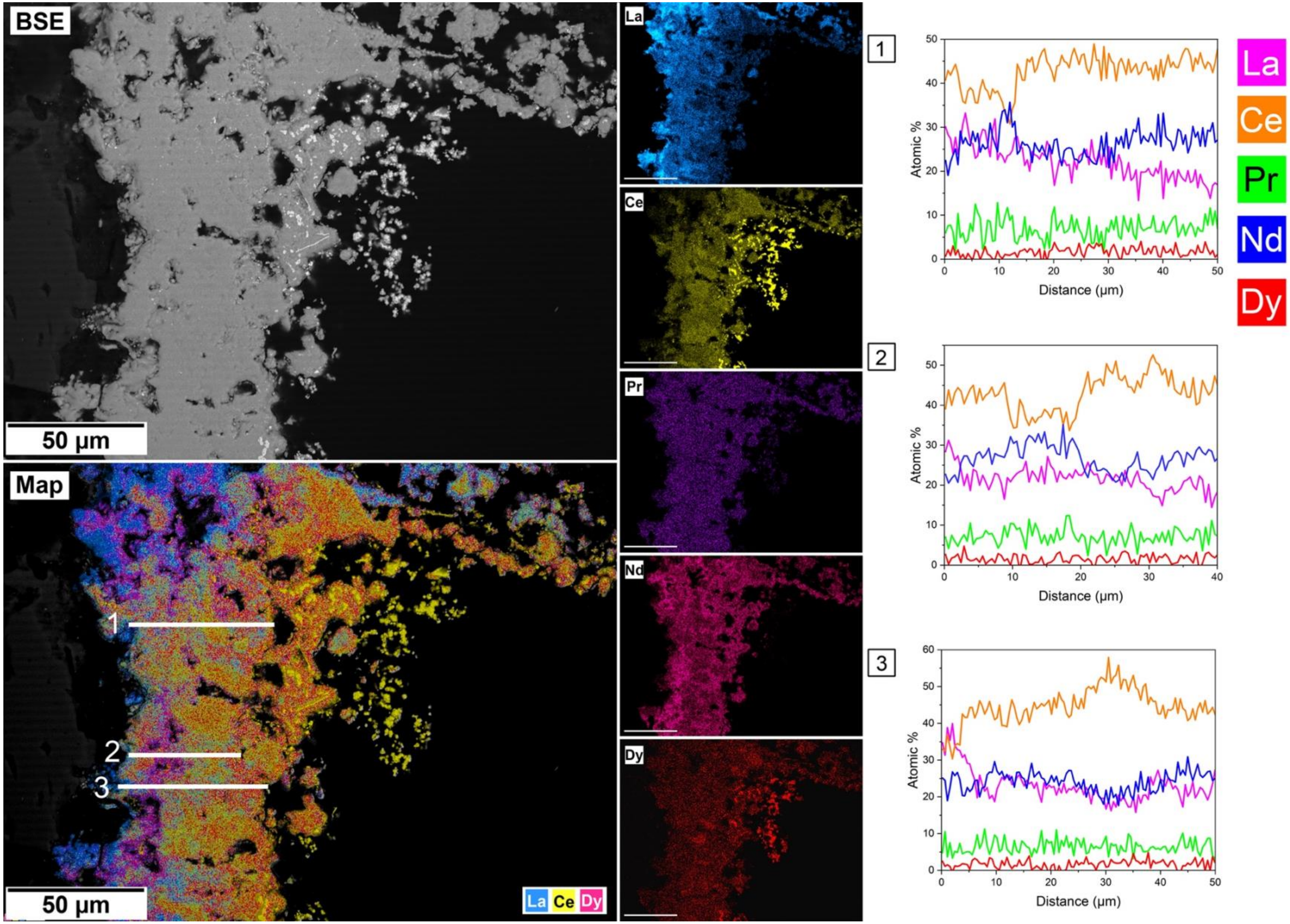

Supplement: Supplementary file 1 — Supporting Information [file GCH2-8-2400074-s001.pdf]
